# Two Pregnancy Care Models in Poland—A Descriptive–Comparative Study

**DOI:** 10.3390/clinpract13050103

**Published:** 2023-09-20

**Authors:** Marta Gallas, Aleksandra Gaworska-Krzemińska, Katarzyna Pogorzelczyk

**Affiliations:** 1 Institute of Nursing and Midwifery, Department of Nursing Management, Medical University of Gdansk, M. Sklodowskiej-Curie Street 3a, 80-227 Gdansk, Poland; aleksandra.gaworska-krzeminska@gumed.edu.pl; 2 Independent Researcher, Skødstrup, 8541 Aarhus, Denmark

**Keywords:** pregnancy, care, quality, model, midwife, satisfaction

## Abstract

Care for a pregnant woman can take various organizational forms. The World Health Organization (WHO) recommends leaders increase research into health systems. The aim of this is to manage the healthcare system in such a way as to provide beneficiaries with access to high-quality services with limited financial resources. The study presented in this paper was conducted using a diagnostic survey on a group of 1697 Polish women in the traditional model of care (TM) and 3216 women in the Coordinated Care for Pregnant Women Program (CCP). Two research tools were used in this study. The first is a survey prepared by the National Health Fund, the second is an author’s survey. The results indicate that most women (85%) receiving care under the CCP received effective pain management, compared to 67% under the traditional care model (*p* < 0.001). In the CCP, women were significantly more likely to receive midwife assistance in infant care (90%) than women in the traditional care model (60%) (*p* < 0.001). Significantly more CCP patients want to return to the same hospital for their subsequent childbirth (74%) than women in the traditional model of care (43%) (*p* < 0.001). In patients’ opinion, the new CCP model is superior in meeting their needs and providing higher-quality services. However, educating women that an obstetrician-gynecologist and a midwife can manage their pregnancy is still necessary. In addition to the CCP program, special attention should be paid to available pain management during childbirth since the use of analgesia is still insufficient in Polish hospitals.

## 1. Introduction

One of the goals set out by the UN Human Rights Council is to improve the quality of perinatal care in the world. This assumption is also confirmed in declarations relating to international politics [1,2]. The goal of sustainable development is to eliminate perinatal deaths that could have been prevented by reducing the inequalities in access to health services and improving their quality [3]. The World Health Organization (WHO) recommends leaders increase research into health systems. The aim of this is to manage the healthcare system in such a way as to provide beneficiaries with access to high-quality services with limited financial resources [4]. According to international studies conducted in 57 countries [5], an increase in the number of antenatal educational visits is associated with a reduced risk of neonatal death [6]. In addition, studies show that improving the quality of perinatal care contributes to reducing the infant mortality rate by 23%, and it should also be noted that clinical outcomes are impacted by extra-clinical factors of healthcare, such as the quality of medical services [7].

In Poland, every pregnant woman can choose one of two models of pregnancy care. In the first model (traditional model, TM), a woman makes separate choices of a doctor, a midwife, and a hospital where she will give birth. Each medical professional can work in a different healthcare facility, and they are not likely to contact each other [8,9]. The second model is the Coordinated Care for Pregnant Women Program (CCP), introduced in Poland in 2017. In this model, a woman chooses the hospital for labor, and then she chooses her team of healthcare professionals consisting of a gynecologist-obstetrician, midwife, and other staff from among the hospital staff. The hospital of choice is the unit coordinating care for the pregnant woman. Once she joins the CCP, a patient cannot receive care in other healthcare units. The choice of CCP or TM is voluntary. When a woman decides to join the CCP, she must sign an agreement in which she is obliged to use the services of the facility that implements the CCP only; it is not possible to use the TM in entities that do not implement the CCP. By signing the contract for the implementation of the CCP, the hospital cannot provide care for a pregnant woman under the TM at all. The purpose of the CCP is to provide comprehensive care for a pregnant woman and, in case of obstetric adverse events, early diagnosis and appropriate treatment. Hospitals with at least 600 births annually are eligible to join the program [10,11]. The schedule of visits and examinations required for both models are the same.

The main tasks of a person taking care of a pregnant woman include, in particular [12]:

- Assessment of the health of the pregnant woman, fetus, and newborn.
- Detection and elimination of pathology risk factors.
- Control of the patient’s test results, including blood group determination.
- Conducting childbirth with a focus on natural childbirth.
- Taking care of the pregnant woman, midwife, and newborn.
- In the event of indications, providing the woman with specialist care.
- Support for the mother and her relatives during childbirth and the postpartum period.
- If necessary, ensure a faster termination of pregnancy.

When pathological symptoms appear, when a midwife takes care of a pregnant woman, she must immediately be under the care of an obstetrician.

According to applicable recommendations in Poland, pregnant women should have eight check-ups with a physician or midwife. If a midwife provides care during pregnancy, a physician should be consulted three times: up to week 10, between 24 and 26 weeks, and between 38 and 39 weeks. The remaining five visits can be scheduled with the midwife. A pregnant woman should have eight follow-up visits with a doctor or midwife. Even when the care of a pregnant woman is provided by a midwife, medical consultation should take place three times: up to the 10th week of pregnancy, between the 24th and 26th week of pregnancy, and between the 38th and 39th week of pregnancy. The remaining five visits can be made by a midwife. The doctor performs the first- and second-trimester morphological ultrasound and the screening for aneuploidy. In the case of physiological pregnancy, the patient should be followed up at least every 3–4 weeks. Physicians monitor 99% of pregnancies in Poland. Within the framework of perinatal care, the midwife is in charge of antenatal education, which can be carried out individually or in groups. Individual antenatal education is also the task of the obstetrician-gynecologist (OB/GYN). Education covers the period of pregnancy, childbirth, and the postpartum period. In Poland, patients usually only see the midwife before an appointment with their OB/GYN. Significantly, between weeks 21 and 31, a pregnant patient can visit the midwife once a week, and as of week 32, until labor—twice a week within the framework of the National Health System. During these visits, the midwife conducts perinatal education instead of birthing school, and the women do not use that solution [12,13].

Nursing care for the baby and the mother is the midwife’s responsibility. The midwife should mark the newborn, cut the umbilical cord, and assess the newborn on the Apgar scale; if possible, this assessment should take place on the mother’s belly [12].

Before the mother and child are discharged from the hospital, external medical documentation of the newborn should be prepared, which will include, among other things, recommendations on the child’s nutrition, instructions on how to latch the child to the breast, and information on the need to choose a primary care midwife who will perform patronage visits [12].

Breastfeeding information should be consistent and up-to-date, and instruction should be given on proper latching on and a variety of feeding positions.

The midwife’s tasks during patronage visits specifically include: assessment of the general condition of the patient, the condition of the uterine muscles, perineal wounds, and the condition of the mammary glands and nipples. The midwife should also assess lactation, body hygiene, and the woman’s mental state. The midwife’s tasks also include checking the general condition of the newborn, the condition of its skin, reflexes, nutrition, eyes, the condition of the umbilical cord stump, how to care for body hygiene, and the hygiene and safety of the room in which the newborn is staying. They should also pay attention to family relationships and care possibilities of the family, encourage natural feeding, inform about the need to take the child for vaccinations, and inform about the need to visit an obstetrician in the eighth week after delivery [12].

In both groups, the tasks of the midwife before and after childbirth are the same. Patients decide whether they want to be part of the CCP or TM at the first visit to the doctor or midwife. The medic presents the patients with both options.

This study aims to compare the quality parameters of two models of care for pregnant women in Poland: the CCP and TM models. The study compared qualitative parameters related to childbirth, such as labor pain relief, help provided by midwives with the baby after birth, and privacy respect.

## 2. Materials and Methods

A schematic of the carried out works to collect data required for the purpose of the research can be found in Appendix A.

The study design was cross-sectional. In the first half of 2019, all 27 hospitals implementing the CCP were asked to provide data based on patient surveys evaluating this model. Eight hospitals (33%) located throughout Poland agreed to share completed surveys. Hospitals provided data for over 1000 women for each of the years between 2017 and 2019. Received questionnaires were rewritten into MS Excel, for comparison. In the second half of 2019, further data were collected directly from hospitals. Data were collected from 3216 patients in total. Patients provide informed consent to complete the postpartum survey by joining the program. In the next step, we created a proprietary research tool based on an earlier survey developed by the National Health Fund (Polish public health insurer). Private healthcare was available to women in both groups, while only public healthcare was considered in the study. The author’s questionnaire also contained sections on outpatient care, perinatal care, and postpartum care. In addition, the first part of the survey included the metric part, which was missing in the survey prepared by the National Health Fund. The survey did not require translation. This tool was shared online on sites related to perinatal care. The survey was only in electronic form. The link was available for two months, and filling in the survey was voluntary. The survey was available online for 2 months. It was impossible to complete the survey without completing all the questions, thanks to which all the obtained surveys were complete. When one of the respondents interrupted the survey in the middle, the survey was not saved on the disk. The study involved 1697 women in traditional care. The inclusion criterion was childbirth within six months of the study. If a woman was pregnant more than once, only the last pregnancy was taken into account. The exclusion criteria included childbirth more than six months prior to the study and pregnancy management under the CCP. If there was a non-response error, the survey was not taken into account. In the case of online surveys, this was not possible.

The questionnaire used for CCP patients consisted of three parts: A, S, and P. The first (A) concerned outpatient care and consisted of eight single-choice questions. The second part (S) focused on care in the hospital, which contained thirteen single-choice questions, and the third part (P) assessed care during the puerperium, which consisted of four single-choice questions.

As researchers comparing both models, we had no influence on the research tool for the study of women in the CCP model (National Health Fund decision), which did not include respondents’ demographic and social data. We present patient data only from the traditional model because we studied them with our proprietary tool, which included additional questions.

The study was conducted with ethical principles. The Bioethics Committee of the Medical University of Gdansk approved the study. The study was conducted under the Declaration of Helsinki. The collected data were stored on a password-protected online drive with two-step verification, to which only the first researcher had access. Before participating in the study, each participant had to consent to take part, and the study was voluntary.

### Statistical Analysis

Statistical analysis was performed in IBM SPSS Statistics 28. Chi-square independence tests were calculated to assess the relationships between categorical qualitative variables. The factor Phi was used to measure the relationship in the 2 × 2 tables. (The contingency coefficient Phi is a measure of dependence determined for 2 × 2 contingency tables. The value of the coefficient Phi is in the range <−1, 1>. The closer this value is to −1, the weaker the relationship between the examined features, and the closer to 1, the stronger.) Cramer’s V (*V_c_*) was used for larger tables (m × n, where m and/or n > 2: relationships between two nominal variables, with at least one that takes more than two values). These measures take values in the range of 0 to 1, where 0 means no relationship and 1 signifies a full relationship between the variables. In this range, the significance level is α = 0.05, obtained from the chi-square test.

## 3. Results

The statistical analysis of the results obtained from the patients in the traditional model indicates that the largest group was women aged 26–30 years (52%) with higher education (80%) living in cities with populations over 50 thousand (53%). The second most numerous group comprised women aged 31–35 years (28%) with higher education (90%) living in cities with populations over 50 thousand (63%). The least numerous group was women over 40 years of age (0.5%) with higher education (75%) living in the countryside (38%) and cities with populations up to 50 thousand (38%). Table 1 presents the details.

Further analyses concern both groups. As shown in Table 2 under the CCP, patients were more likely (92%) to decide as early as at the beginning of their pregnancy whether they wanted a doctor or a midwife to manage their pregnancy than patients receiving care under the standard model of care (26%).

A chi-square independence test was performed to analyze the differences in the choice of healthcare professional between CCP and TM patients. The results are shown in Table 3.

The analysis revealed significant differences in the availability of the choice of the pregnancy caregiver between CCP and TM patients. Therefore, to understand this relationship, additional post hoc analyses were performed to further explore the issue (the results are presented in Table 3). It turned out that most women who had the opportunity to choose their pregnancy caregiver participated in the CCP.

**Figure 1:**
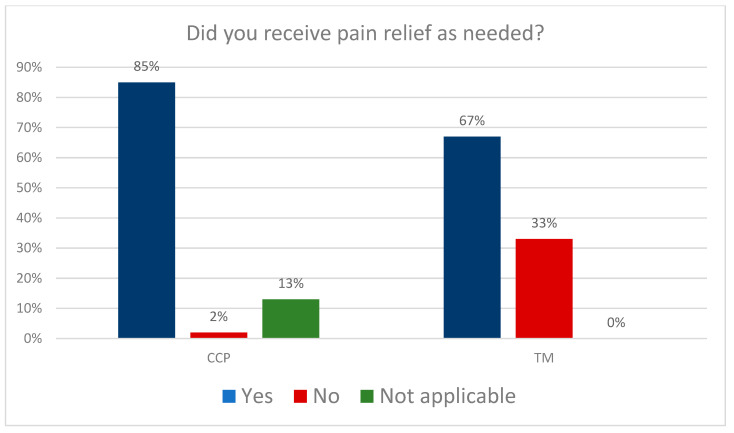
Pain management.

Next, we analyzed the data on pain management. Figure 1 shows the results: The percentage of patients who received effective pain treatment when needed was 85% in the CCP group and 67% in the TM group.

A chi-square independence test was performed to analyze the differences in effective pain management between CCP and TM patients. The results are presented in Table 4.

The analysis showed significant differences in effective pain management between patients receiving coordinated care (CCP) and patients under the traditional model of care (TM). Therefore, additional post hoc analyses were performed to further explore this issue (the results are presented in Table 4). The results indicated that most women who evaluated pain treatment as effective were in the CCP group.

The next question concerned the care provided by midwives. As shown in Table 5, CCP patients were much more likely to receive midwife assistance in child childcare (90%) than TM patients (60%). Table 6 presents the details.

A chi-square independence test was conducted to test the differences between CCP and TM patients in the assistance they received in childcare. The results are presented in Table 6.

There were significant differences between CCP and TM patients in the assistance received in childcare activities. Additional post hoc analyses were performed to further explore this issue (the results are presented in subscript in Table 6). They indicated that most women who received assistance in childcare were in the CCP group.

**Figure 2:**
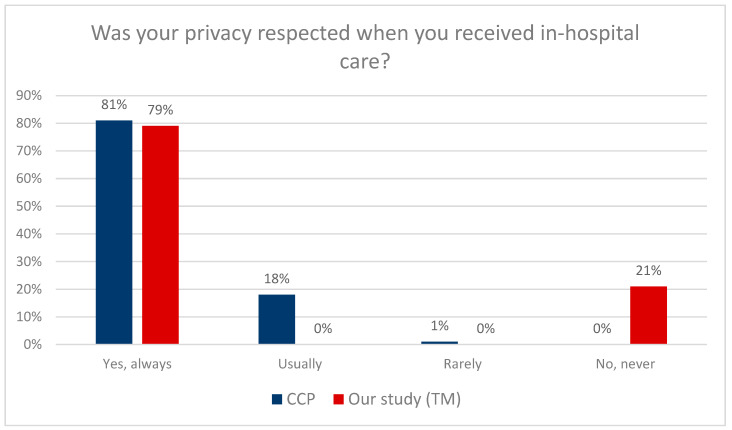
Respecting privacy during hospital stay.

As shown in Figure 2, both the CCP (81%) and TM (79%) patients reported that their privacy was respected in the hospital. Significantly, the surveys used different scales: there were four response options in the CCP: yes, always, usually, rarely, no, and never, while the TM survey used a two-step scale: yes and no. Consequently, statistical analysis was not possible.

**Figure 3:**
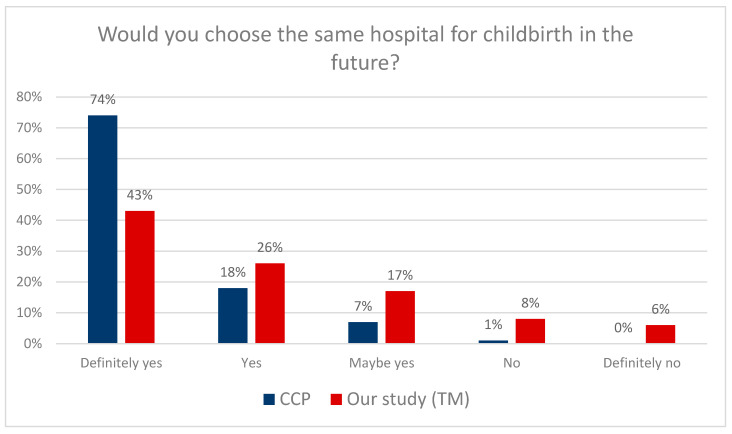
Choosing the same hospital for labor in the future.

The next question concerned the willingness to return to the same hospital for subsequent childbirth. According to the data presented in Figure 3, significantly more CCP patients will decide to choose the same hospital for childbirth again (74%) than TM patients (43%). In addition, women who received care from a midwife expressed high satisfaction with pregnancy management (greater than in the case of a physician as a caregiver) (*p* = 0.001, χ^2^ = 627.12). Table 7 presents detailed data.

Then, an independent chi-square test was performed to analyze the differences in the willingness to return to the same maternity facility between CCP and TM patients.

There were significant differences in the willingness to choose the same maternity facility between CCP and TM patients. Additional post hoc analyses were performed to further explore this issue (the results are presented in subscript in Table 7). The results indicate that the majority of women who would definitely choose the same maternity facility again received care under the CCP.

## 4. Discussion

Research on the quality of perinatal care in Poland has been ongoing for years. Pregnancy is undoubtedly one of the most important events in a woman’s life, so paying attention to the above problem seems necessary. It is vital for a woman to choose who will be the pregnancy caregiver. However, according to the results of our study, patients who received care within the Coordinated Care for Pregnant Women (CCP) could choose who would manage their pregnancy (physician or midwife) more often (92%) than patients receiving care from the traditional model of care (TM), where only 26% of women could make such a decision. This indicates that the traditional model (TM) is less likely than the CCP to provide pregnant women with the choice of their caregiver. In his speech, the Ombudsman in Poland drew particular attention to the issue of availability of choice in selecting the obstetrician provider according to the patient’s gender preference. For some women, receiving care from a woman or a man is essential. Women should be given a choice, especially if they have experienced sexual violence [13]. A study by Susannah Brady, Nigel Lee, Kristen Gibbons, et al. focused on the women-centered aspect of midwifery care; the authors analyzed midwifery care during delivery. It has been noticed that women who receive timely and accurate information related to the progress of labor are able to make informed decisions. Research has suggested that midwives should help women adjust their expectations to the current state of labor and real events. Midwives should empower women’s choices while alleviating fear and anxiety and building women’s self-confidence. A large aspect was also attached to the continuity of care, partner relationship with the midwife, and the education of women and the individual midwife. All of the above aspects are the basis of the CCP introduced in Poland [14].

Much of the research in this review confirms that the concept of woman-centered care is the foundation of good midwifery practice for midwives to act as anchoring companions, listening to the woman, allowing her to participate in her care, and building trusting relationships.

Another vital element of perinatal care is the availability of anesthesia during labor. One of the most common fears of women associated with pregnancy and childbirth is pain during labor [15]. There are available forms of non-pharmacological and pharmacological pain relief. The pharmacological forms include epidural anesthesia, pain control gases, and intravenous opioids. The non-pharmacological pain relief methods include water birth and immersion, transdermal electrical nerve stimulation (TENS), aromatherapy, acupuncture and acupressure, and massage techniques [16,17]. In 2016, the Childbirth with Dignity Foundation (Fundacja Rodzić po Ludzku) monitored all hospitals in Poland. According to the data obtained, as part of non-pharmacological labor pain relief, 98% of the hospitals surveyed offer breathing exercises, 97%—baths or showers, 86%—massages, and approx. 50%—hot compresses, while only 38% of hospitals can provide patients with transdermal electrical stimulation TENS [18,19]. However, according to the Supreme Audit Office (SAO), only 64% of the surveyed women were asked if they wanted to change positions during labor, only 54% were offered to perform breathing exercises, 45% were encouraged to walk, and only 34% of women in labor were asked if they wanted to shower. In fact, only 30% of women used non-pharmacological pain relief [20,21]. As part of pharmacological pain relief, hospitals most often declare access to inhaled analgesia—84%, epidural anesthesia—49%, and only 30% provided pethidine. According to patients’ declarations, inhalation analgesia was used by 51%, epidural anesthesia—by 40%, and pethidine—by only 13% of patients in labor. Epidural anesthesia was the most common—it is available in 83% of referral level III hospitals and only in 36% of level I hospitals. Hospitals indicated an insufficient number of anesthesiologists as the major problem with access to epidural anesthesia [22]. Both of them should be offered in the models of care studied. The choice of epidurals or alternative methods should be up to the patient. One of the aims of this study was to compare the quality parameters of both models of care in terms of alleviating labor pain, and 85% of CCP patients and 67% of TM patients received effective pain treatment. In the UK, 30% of women in labor use epidural anesthesia; the percentage in Scotland is 22% and is as high as 73% in the United States. In 2017, a study was conducted in India among doctors and patients of the Obstetric Department of a University Affiliates Hospital on the availability and use of anesthesia during labor. More than half of the respondents (59%) wanted to receive epidural anesthesia during labor, while only 13% received it [23,24]. In Ireland, women identified access to anesthesia as the most important aspect of choosing a maternity facility [25]. The use of anesthesia during labor is associated with higher rates of patient satisfaction and increased willingness to become pregnant again [18,26]. However, according to a study conducted in Poland in 2018, there is still room for improvement in educating women about available labor pain relief methods [27]. Epidural anesthesia is still the gold standard in pain relief.

The regulation of the Minister of Health of 16 August 2018 on the organizational standard of perinatal care clearly states that a woman should receive education on breastfeeding, lactation support, and solving lactation problems in the framework of antenatal education. During childbirth, she should receive instruction on latching and support in breastfeeding. The midwife should also supervise the first breastfeeding session and provide the mother with consistent and up-to-date information [18]. Studies show that women who receive support from a midwife during childbirth are significantly more likely to be successful in breastfeeding for the recommended six months of a baby’s life than those who only visit a physician during pregnancy. This study aims to compare the quality parameters of two models of care regarding a midwife’s assistance with the child after birth. Both the CCP patients (81%) and the TM patients (79%) received assistance from the midwife in childcare. What is important, a report by the Center for Lactation Science noted that more than half of midwives do not have up-to-date knowledge of recommendations for natural feeding. The solutions midwives suggested to remedy feeding problems were more straightforward but incorrect [20].

A woman giving birth should be treated with respect and should be allowed to make decisions about herself and her newborn. The hospital staff should pay attention to the speech, both verbal and non-verbal, tone of voice, words, body posture, and eye contact. Personnel should ask the mother about her needs and expectations and, if possible, realize them. Each person caring for the mother should introduce themselves and explain their role in caring for her. Staff should have a calm and trustworthy attitude. Privacy and the feeling of giving birth should be respected. Persons taking care of a woman in labor should familiarize themselves with her birth plan and, if possible, implement it, presenting the woman with the possibilities of pain relief, the use of physical activity, and optimal positions during childbirth, and call for help. The patient has the right to relieve labor pain, including using non-pharmacological methods such as physical activity, taking appropriate positions with available equipment, breathing techniques, massages, water immersion or acupressure, and pharmacological treatments [28,29].

Western governments are paying increasingly more attention to the quality of non-clinical services when monitoring the performance of the healthcare system [30,31]. In Albania, a study was conducted on the non-clinical aspects of healthcare affecting the quality of services provided. The study clearly showed that patient involvement in aspects related to their health is essential, as is paying particular attention to patient autonomy. Respondents highly rated their experience with the quality of healthcare that was focused on the conversation with the patient [32].

Also, the study conducted at the Mayo Clinic indicated the importance of interactions with clinical staff and their impact on the treatment process. The observations indicate that the staff members who contact patients directly should be thoroughly screened during the recruitment process to ensure that their behavior and the values they represent are consistent with the values of the medical institution. Staff should participate in ongoing training to enhance interpersonal skills, such as creativity, productivity, empathy, and extra effort in non-clinical support services that can influence and shape patients’ and their families’ perception of the overall quality of services [33].

In its latest guidelines, “Intrapartum care for a positive childbirth experience”, The World Health Organization (WHO) pays special attention to providing respectful maternity care that reduces maternal morbidity and mortality. An individual approach to each patient and effective communication were considered very important. Positive childbirth experiences were considered the most important aspect [33]. Future maternity decisions are undoubtedly influenced by the stress experienced during labor—it has been observed that previous negative birth experience leads to delaying subsequent pregnancy and requesting a cesarean section during the next labor [33,34]. According to the presented analysis results, far more CCP patients (74%) than TM patients (43%) decide to choose the same hospital again for labor. The report prepared by the Childbirth with Dignity Foundation in 2018, based on monitoring maternity wards, showed several irregularities related to the care of a pregnant woman, such as disrespectful and offensive behavior on the part of medical personnel towards women in labor [35,36]. Professional medical care and respect for the patient’s dignity should be a pillar of medical care [37,38,39]. The SAO report did not provide favorable data either—hospitals did not protect patient’s privacy during childbirth; in 10 out of the 16 studied hospitals, delivery rooms and physicians’ offices were arranged in such a way that when the doors were opened, bystanders could see a woman in an intimate situation. One of the aims of the study was to compare the quality parameters of both models of care in terms of maintaining patients’ privacy during care. In this study, both the CCP (81%) and TM (79%) patients reported that their privacy was respected in the hospital. The SAO audit was carried out in 2016, which suggests that the care for pregnant women has improved in this area [38]. For example, in Chile, only 56% of women felt supported and respected by medical staff [40,41]. In the UK, on the other hand, when respondents were asked if they felt respected by midwives and other medical staff, 82% of respondents indicated a positive answer [42]. However, in a similar study conducted among women with disabilities, unfortunately, more than half of them—56%—indicated that providers did not treat them with the required respect in maternity care [43].

## 5. Conclusions

1. The patients’ opinions indicate that the new Coordinated Care for Pregnant Women program (CCP) protects their needs to a greater extent, allows for greater opportunity to make independent decisions, and provides services of a higher quality.
2. It is necessary to continue to promote pregnancy management not only among OB/GYNs but also midwives.
3. In addition to the CCP program, special attention should be paid to available pain management in labor since the use of analgesia is still insufficient in Polish hospitals.
4. The CCP program was considered more satisfactory by patients—women received effective pain treatment more often, they could rely on help from midwives more often after childbirth, and most of them would choose the same hospital again for childbirth.
5. There were no data regarding women’s marital status or place of living to provide additional information and check if there is any relationship between choosing each model. From interviews, we found that ladies chose their model out of curiosity, recommendation, or could not decide and chose one randomly.

## 6. Limitations

A limitation of the work was the use of retrospective data and the inability to obtain demographic data from patients receiving care in the CCP, as we used a secondary source.

Another limitation was the inability to compare our results with other results of patients using the CCP because no one has conducted such studies in Poland yet, and the models of foreign care differ from those available in Poland. There is therefore a need for more research.

The next limitation was access to the questionnaires. They were only available online, so women who did not use the Internet were not able to fill out the questionnaire.

There were no data regarding women’s marital status or place of living to provide additional data and check dependence.

## Figures and Tables

**Table 1:** Characteristics of the respondents from the traditional model.

Age Group	*N*	% Per Group	Place of Residence	*N*	% Per Group	Education	*N*	% Per Group
18–25	254	14.98%	countryside	100	39.37%	primary	5	1.97%
			town with up to 50,000 inhabitants	53	20.87%	secondary	125	49.21%
			city of over 50,000 inhabitants	101	39.76%	vocational	8	3.15%
						higher	116	45.67%
26–30	885	52.18%	countryside	248	28.02%	primary	0	0.00%
			town with up to 50,000 inhabitants	171	19.32%	secondary	170	19.21%
			city of over 50,000 inhabitants	466	52.66%	vocational	10	1.13%
						higher	705	79.66%
31–35	469	27.65%	countryside	97	20.68%	primary	0	0.00%
			town with up to 50,000 inhabitants	77	16.42%	secondary	46	9.81%
			city of over 50,000 inhabitants	295	62.90%	vocational	1	0.21%
						higher	422	89.98%
30–40	80	4.72%	countryside	10	12.50%	primary	0	0.00%
			town with up to 50,000 inhabitants	18	22.50%	secondary	8	10.00%
			city of over 50,000 inhabitants	52	65.00%	vocational	0	0.00%
						higher	72	90.00%
40+	8	0.47%	countryside	3	37.50%	primary	0	0.00%
			town with up to 50,000 inhabitants	3	37.50%	secondary	2	25.00%
			city of over 50,000 inhabitants	2	25.00%	vocational	0	0.00%
						higher	6	75.00%

**Table 2:** The decision concerning the choice of the professional managing the pregnancy (midwife or doctor).

Responses	CCP	Traditional Model (TM)
Yes	2962 (92%)	4212 (26%)
No	250 (8%)	1221 (74%)
Total	3212 (100%)	1642 (100%)

**Table 3:** Results of the chi-square analysis for the differences in the choice of the healthcare professional between CCP and TM patients.

		Group				Total		χ^2^	*p*	Ф
		CCP (Coordinated Care for Pregnant Women)		TM (Traditional Model)						
		*N*	%	*N*	%	*N*	%			
The decision concerning the choice of the professional managing the pregnancy	no	255 _a_	7.9%	1221 _b_	74.4%	1476	30.4%	2270.54	<0.001	0.68
	yes	2962 _a_	92.1%	420 _b_	25.6%	3382	69.6%			
Total		3217	100.0%	1641	100.0%	4858	100.0%			

χ^2^—the result of the chi-square test; *p*—the significance of the chi-square test; Ф—the strength of the effect; each letter in the subscript indicates a subset whose column proportions do not differ significantly from each other at the level of 0.05.

**Table 4:** Chi-square analysis results for the differences in effective pain management between CCP and TM patients.

		Group				Total		χ^2^	*p*	*V_c_*
		CCP (Coordinated Care for Pregnant Women)		TM (Traditional Model)						
		*N*	%	*N*	%	*N*	%			
Effective pain management	no	73 _a_	2.3%	536 _b_	32.7%	609	12.6%	1054.24	<0.001	0.47
	yes	2730 _a_	84.9%	1105 _b_	67.3%	3835	78.9%			
	not applicable	414 _a_	12.9%	0 _b_	0.0%	414	8.5%			
Total		3217	100.0%	1641	100.0%	4858	100.0%			

χ^2^—the result of the chi-square test; *p*—the significance of the chi-square test; *V_c_*—the strength of the effect; each letter in the subscript indicates a subset whose column proportions do not differ significantly from each other at the level of 0.05.

**Table 5:** Midwives’ assistance.

	CCP	Our Study (TM)
Yes	2892/90%	991/60%
No	42/1%	651/40%
Not applicable	280/9%	0/0%
Total	3214	1642

**Table 6:** Chi-square analysis results for the differences between CCP and TM patients in the assistance they received in childcare.

		Group				Total		χ^2^	*p*	*V_c_*
		CCP (Coordinated Care for Pregnant Women)		TM (Traditional Model)						
		*N*	%	*N*	%	*N*	%			
Assistance in childcare activities	no	45 _a_	1.4%	650 _b_	39.6%	695	14.3%	1370.00	<0.001	0.53
	yes	2892 _a_	89.9%	991 _b_	60.4%	3883	79.9%			
	not applicable	280 _a_	8.7%	0 _b_	0.0%	280	5.8%			
Total		3217	100.0%	1641	100.0%	4858	100.0%			

χ^2^—the result of the chi-square test; *p*—the significance of the chi-square test; *V_c_*—the strength of the effect; each letter in the subscript indicates a subset whose column proportions do not differ significantly from each other at the level of 0.05.

**Table 7:** Chi-square analysis results for the differences in the willingness to return to the same maternity facility between CCP and TM patients.

		Group				Total		χ^2^	*p*	*V_c_*
		CCP (Coordinated Care for Pregnant Women)		TM (Traditional Model)						
		*N*	%	*N*	%	*N*	%			
Choosing the same maternity facility again in the future	definitely not	13 _a_	0.4%	95 _b_	5.8%	108	2.2%	627.12	<0.001	0.36
	no	23 _a_	0.7%	133 _b_	8.1%	156	3.2%			
	maybe yes	212 _a_	6.6%	270 _b_	16.5%	482	9.9%			
	yes	592 _a_	18.4%	434 _b_	26.4%	1026	21.2%			
	definitely yes	2376 _a_	73.9%	709 _b_	43.2%	3085	63.5%			
Total		3216	100.0%	1641	100.0%	4857	100.0%			

χ^2^—the result of the chi-square test; *p*—the significance of the chi-square test; *Vc*—the strength of the effect; each letter in the subscript indicates a subset whose column proportions do not differ significantly from each other at the level of 0.05.

**Figure A1:**
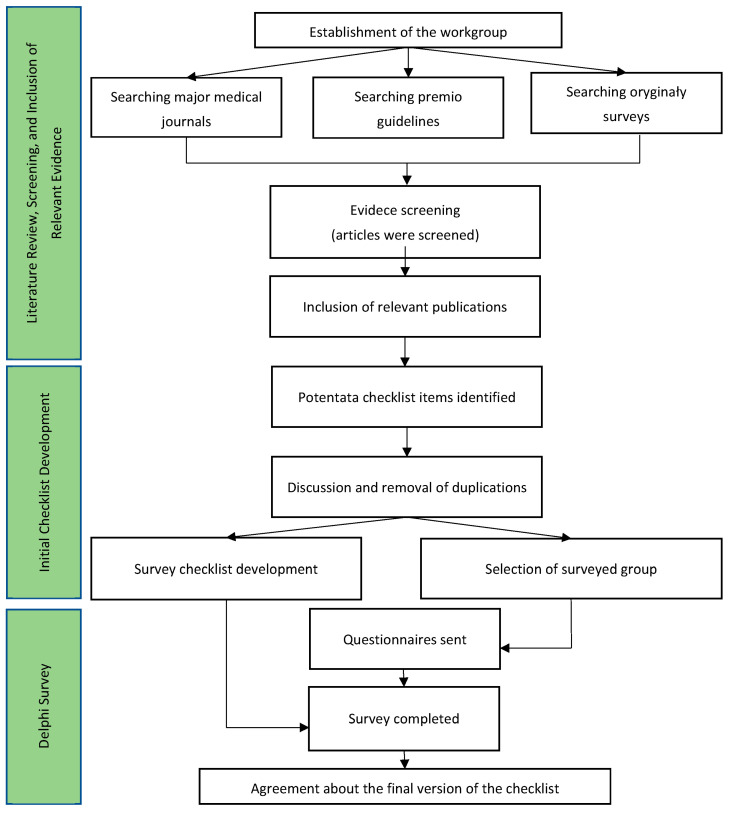
The Workflow Diagram.
